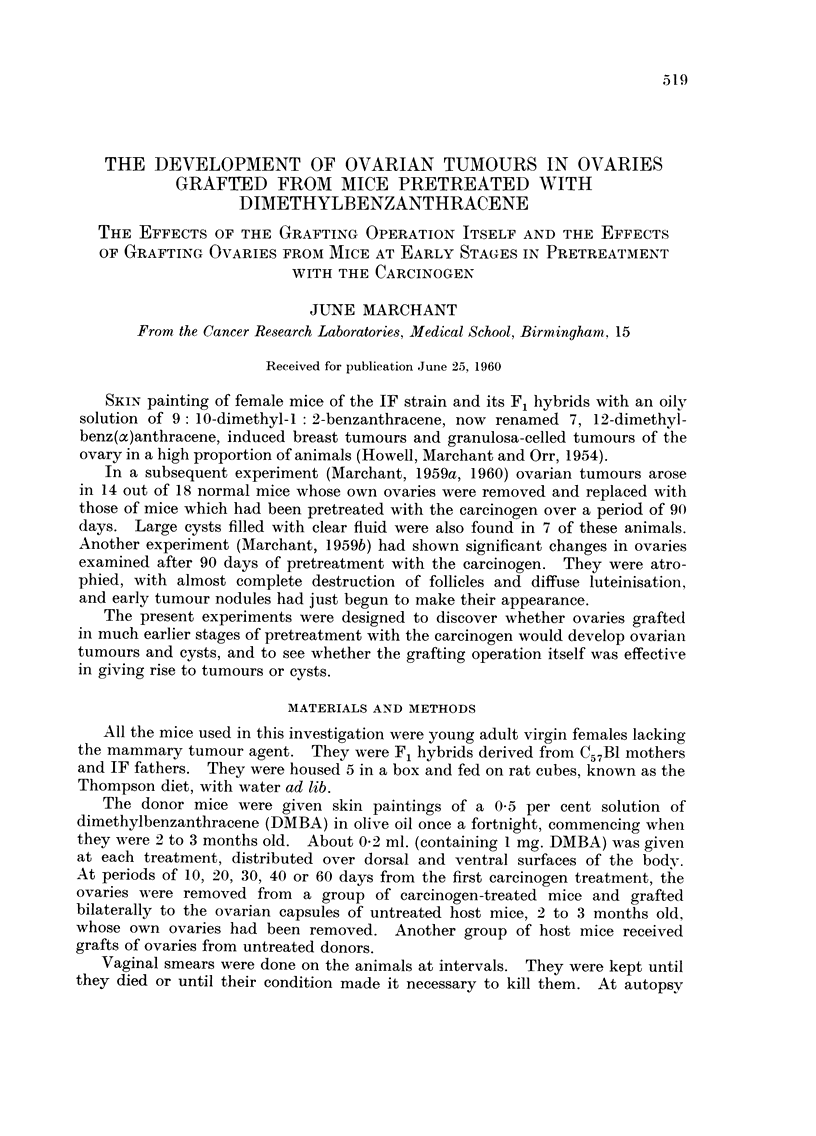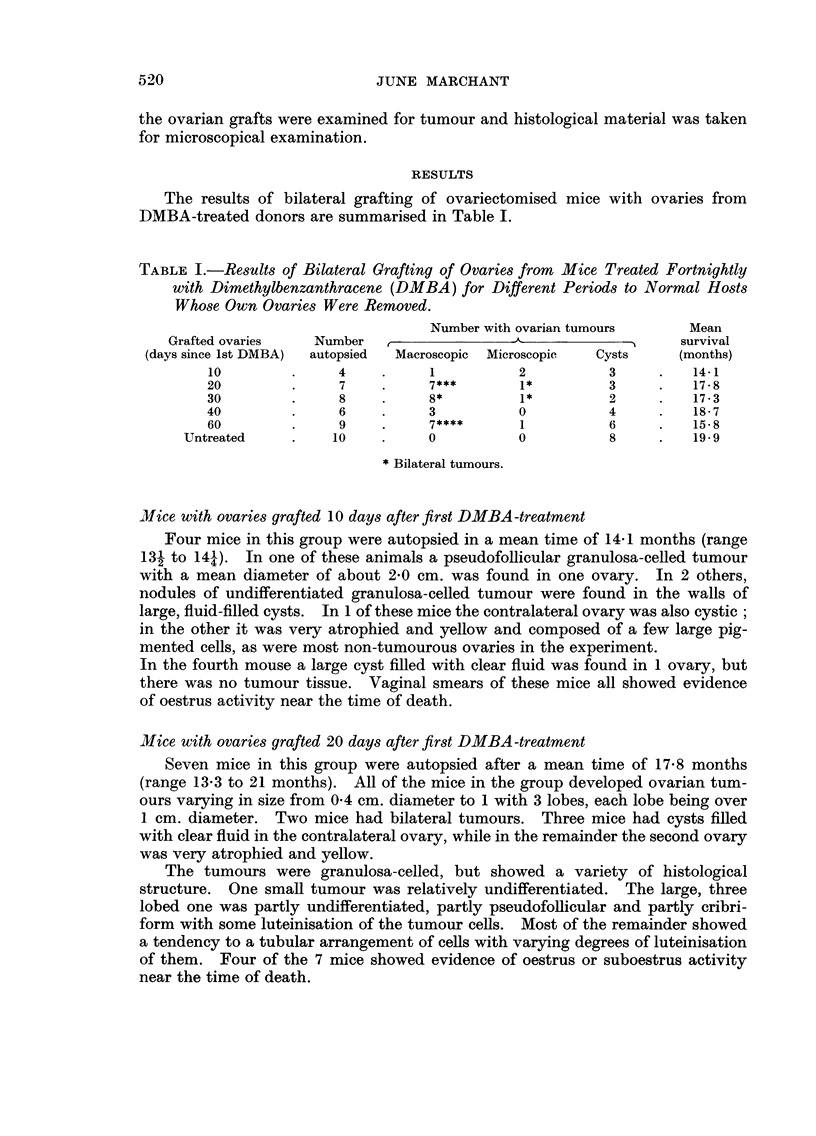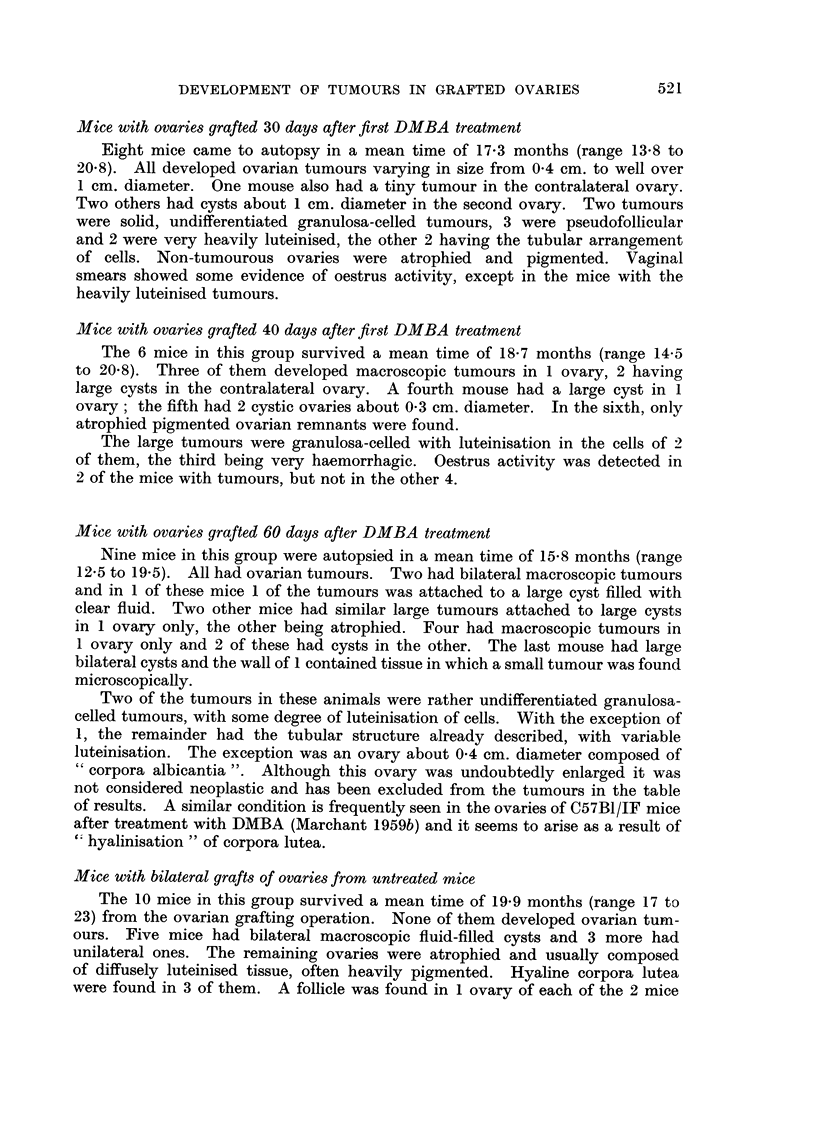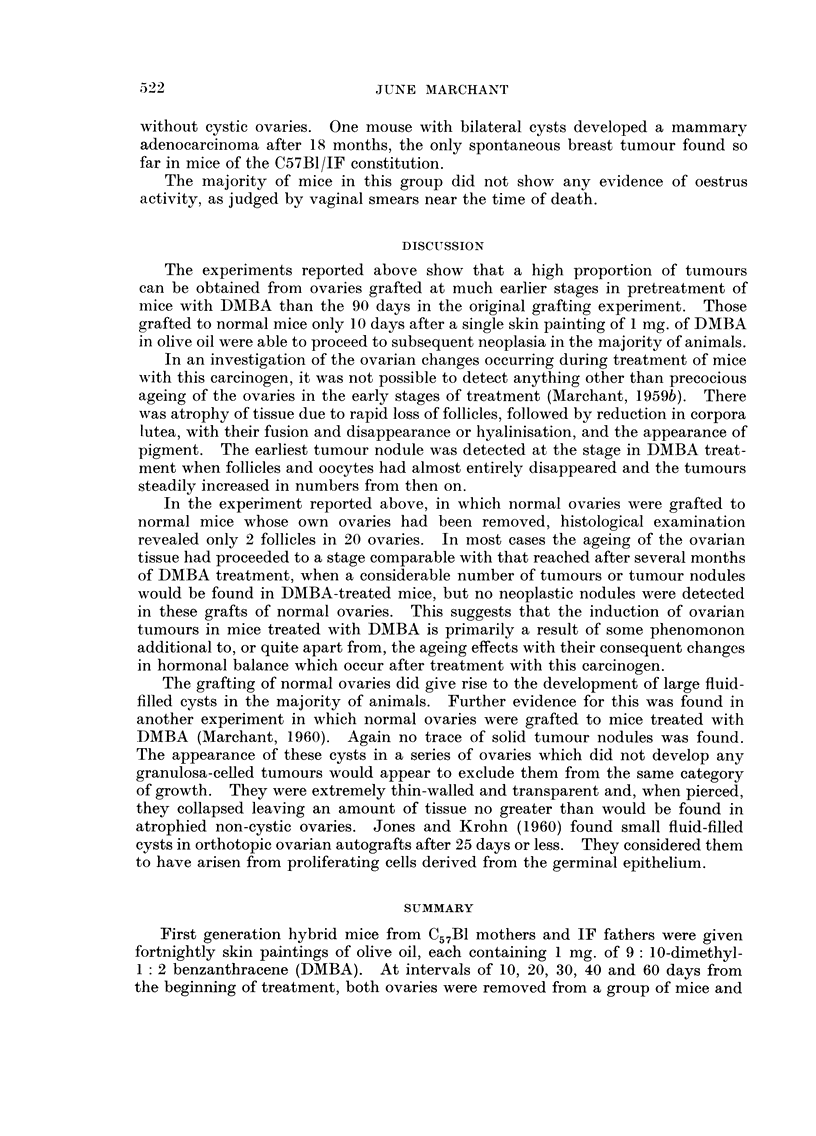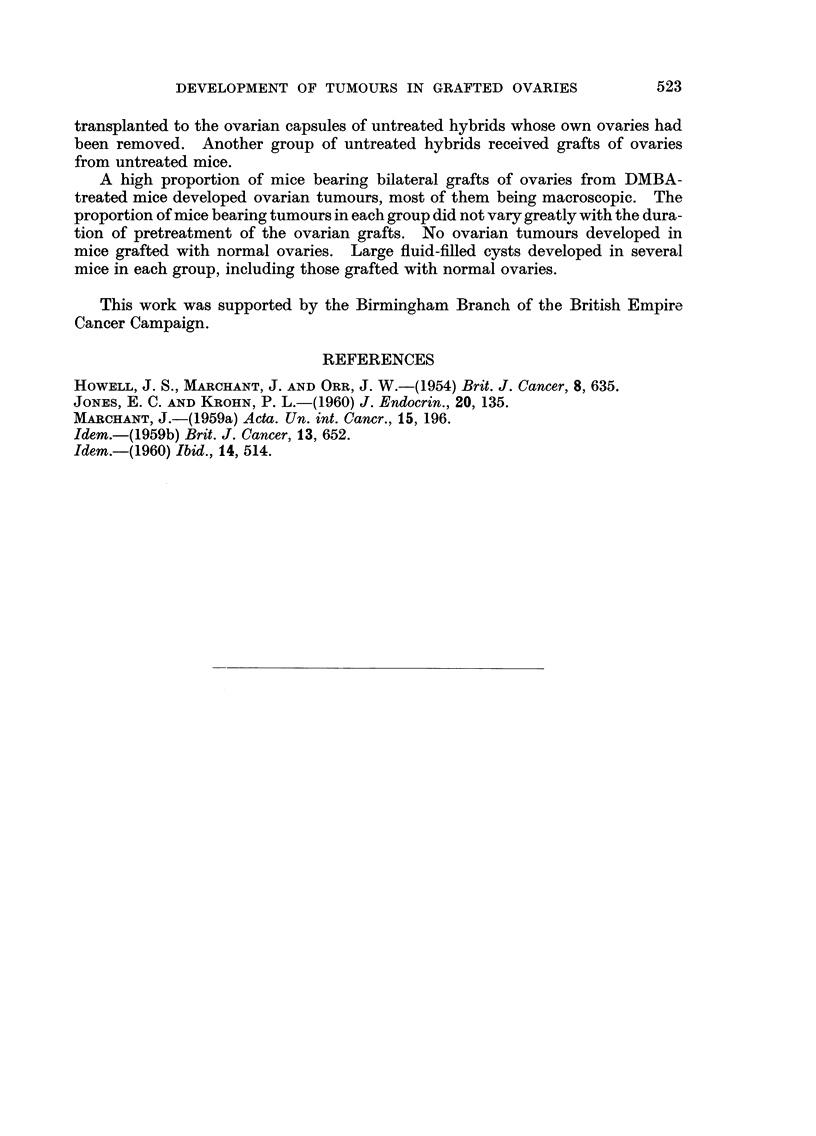# The Development of Ovarian Tumours in Ovaries Grafted from Mice Pretreated with Dimethylbenzanthracene

**DOI:** 10.1038/bjc.1960.58

**Published:** 1960-09

**Authors:** June Marchant


### THE DEVELOPMENT OF OVARIAN TUMOURS IN OVARIES GRAFTED FROM MICE PRETREATED WITH DIMETHYLBENZANTHRACENE

### The Effects of the Grafting Operation Itself and the Effects of Grafting Ovaries from Mice at Early Stages in Pretreatment with the Carcinogen

JUNE MARCHANT

*From the Cancer Research Laboratories, Medical School, Birmingham,* 15

Received for publication June 25, 1960

SKIN painting of female mice of the IF strain and its $F_1$ hybrids with an oily solution of 9 : 10-dimethyl-1 : 2-benzanthracene, now renamed 7, 12-dimethylbenz($\alpha$)anthracene, induced breast tumours and granulosa-celled tumours of the ovary in a high proportion of animals (Howell, Marchant and Orr, 1954).

In a subsequent experiment (Marchant, 1959*a*, 1960) ovarian tumours arose in 14 out of 18 normal mice whose own ovaries were removed and replaced with those of mice which had been pretreated with the carcinogen over a period of 90 days. Large cysts filled with clear fluid were also found in 7 of these animals. Another experiment (Marchant, 1959*b*) had shown significant changes in ovaries examined after 90 days of pretreatment with the carcinogen. They were atrophied, with almost complete destruction of follicles and diffuse luteinisation, and early tumour nodules had just begun to make their appearance.

The present experiments were designed to discover whether ovaries grafted in much earlier stages of pretreatment with the carcinogen would develop ovarian tumours and cysts, and to see whether the grafting operation itself was effective in giving rise to tumours or cysts.

### MATERIALS AND METHODS

All the mice used in this investigation were young adult virgin females lacking the mammary tumour agent. They were $F_1$ hybrids derived from $C_{57}$Bl mothers and IF fathers. They were housed 5 in a box and fed on rat cubes, known as the Thompson diet, with water *ad lib*.

The donor mice were given skin paintings of a 0·5 per cent solution of dimethylbenzanthracene (DMBA) in olive oil once a fortnight, commencing when they were 2 to 3 months old. About 0·2 ml. (containing 1 mg. DMBA) was given at each treatment, distributed over dorsal and ventral surfaces of the body. At periods of 10, 20, 30, 40 or 60 days from the first carcinogen treatment, the ovaries were removed from a group of carcinogen-treated mice and grafted bilaterally to the ovarian capsules of untreated host mice, 2 to 3 months old, whose own ovaries had been removed. Another group of host mice received grafts of ovaries from untreated donors.

Vaginal smears were done on the animals at intervals. They were kept until they died or until their condition made it necessary to kill them. At autopsy the ovarian grafts were examined for tumour and histological material was taken for microscopical examination.

### RESULTS

The results of bilateral grafting of ovariectomised mice with ovaries from DMBA-treated donors are summarised in Table I.

TABLE I.—*Results of Bilateral Grafting of Ovaries from Mice Treated Fortnightly with Dimethylbenzanthracene (DMBA) for Different Periods to Normal Hosts Whose Own Ovaries Were Removed.*

| Grafted ovaries (days since 1st DMBA) | Number autopsied | Number with ovarian tumours | | | Mean survival (months) |
|---|---|---|---|---|---|
| | | Macroscopic | Microscopic | Cysts | |
| 10 | 4 | 1 | 2 | 3 | 14·1 |
| 20 | 7 | 7*** | 1* | 3 | 17·8 |
| 30 | 8 | 8* | 1* | 2 | 17·3 |
| 40 | 6 | 3 | 0 | 4 | 18·7 |
| 60 | 9 | 7**** | 1 | 6 | 15·8 |
| Untreated | 10 | 0 | 0 | 8 | 19·9 |

* Bilateral tumours.

*Mice with ovaries grafted* 10 *days after first DMBA-treatment*

Four mice in this group were autopsied in a mean time of 14·1 months (range $13\frac{1}{2}$ to $14\frac{1}{4}$). In one of these animals a pseudofollicular granulosa-celled tumour with a mean diameter of about 2·0 cm. was found in one ovary. In 2 others, nodules of undifferentiated granulosa-celled tumour were found in the walls of large, fluid-filled cysts. In 1 of these mice the contralateral ovary was also cystic ; in the other it was very atrophied and yellow and composed of a few large pigmented cells, as were most non-tumourous ovaries in the experiment.
In the fourth mouse a large cyst filled with clear fluid was found in 1 ovary, but there was no tumour tissue. Vaginal smears of these mice all showed evidence of oestrus activity near the time of death.

*Mice with ovaries grafted* 20 *days after first DMBA-treatment*

Seven mice in this group were autopsied after a mean time of 17·8 months (range 13·3 to 21 months). All of the mice in the group developed ovarian tumours varying in size from 0·4 cm. diameter to 1 with 3 lobes, each lobe being over 1 cm. diameter. Two mice had bilateral tumours. Three mice had cysts filled with clear fluid in the contralateral ovary, while in the remainder the second ovary was very atrophied and yellow.

The tumours were granulosa-celled, but showed a variety of histological structure. One small tumour was relatively undifferentiated. The large, three lobed one was partly undifferentiated, partly pseudofollicular and partly cribriform with some luteinisation of the tumour cells. Most of the remainder showed a tendency to a tubular arrangement of cells with varying degrees of luteinisation of them. Four of the 7 mice showed evidence of oestrus or suboestrus activity near the time of death.

*Mice with ovaries grafted* 30 *days after first DMBA treatment*

Eight mice came to autopsy in a mean time of 17·3 months (range 13·8 to 20·8). All developed ovarian tumours varying in size from 0·4 cm. to well over 1 cm. diameter. One mouse also had a tiny tumour in the contralateral ovary. Two others had cysts about 1 cm. diameter in the second ovary. Two tumours were solid, undifferentiated granulosa-celled tumours, 3 were pseudofollicular and 2 were very heavily luteinised, the other 2 having the tubular arrangement of cells. Non-tumourous ovaries were atrophied and pigmented. Vaginal smears showed some evidence of oestrus activity, except in the mice with the heavily luteinised tumours.

*Mice with ovaries grafted* 40 *days after first DMBA treatment*

The 6 mice in this group survived a mean time of 18·7 months (range 14·5 to 20·8). Three of them developed macroscopic tumours in 1 ovary, 2 having large cysts in the contralateral ovary. A fourth mouse had a large cyst in 1 ovary ; the fifth had 2 cystic ovaries about 0·3 cm. diameter. In the sixth, only atrophied pigmented ovarian remnants were found.

The large tumours were granulosa-celled with luteinisation in the cells of 2 of them, the third being very haemorrhagic. Oestrus activity was detected in 2 of the mice with tumours, but not in the other 4.

*Mice with ovaries grafted 60 days after DMBA treatment*

Nine mice in this group were autopsied in a mean time of 15·8 months (range 12·5 to 19·5). All had ovarian tumours. Two had bilateral macroscopic tumours and in 1 of these mice 1 of the tumours was attached to a large cyst filled with clear fluid. Two other mice had similar large tumours attached to large cysts in 1 ovary only, the other being atrophied. Four had macroscopic tumours in 1 ovary only and 2 of these had cysts in the other. The last mouse had large bilateral cysts and the wall of 1 contained tissue in which a small tumour was found microscopically.

Two of the tumours in these animals were rather undifferentiated granulosa-celled tumours, with some degree of luteinisation of cells. With the exception of 1, the remainder had the tubular structure already described, with variable luteinisation. The exception was an ovary about 0·4 cm. diameter composed of " corpora albicantia ". Although this ovary was undoubtedly enlarged it was not considered neoplastic and has been excluded from the tumours in the table of results. A similar condition is frequently seen in the ovaries of C57Bl/IF mice after treatment with DMBA (Marchant 1959*b*) and it seems to arise as a result of " hyalinisation " of corpora lutea.

*Mice with bilateral grafts of ovaries from untreated mice*

The 10 mice in this group survived a mean time of 19·9 months (range 17 to 23) from the ovarian grafting operation. None of them developed ovarian tumours. Five mice had bilateral macroscopic fluid-filled cysts and 3 more had unilateral ones. The remaining ovaries were atrophied and usually composed of diffusely luteinised tissue, often heavily pigmented. Hyaline corpora lutea were found in 3 of them. A follicle was found in 1 ovary of each of the 2 mice without cystic ovaries. One mouse with bilateral cysts developed a mammary adenocarcinoma after 18 months, the only spontaneous breast tumour found so far in mice of the C57Bl/IF constitution.

The majority of mice in this group did not show any evidence of oestrus activity, as judged by vaginal smears near the time of death.

### DISCUSSION

The experiments reported above show that a high proportion of tumours can be obtained from ovaries grafted at much earlier stages in pretreatment of mice with DMBA than the 90 days in the original grafting experiment. Those grafted to normal mice only 10 days after a single skin painting of 1 mg. of DMBA in olive oil were able to proceed to subsequent neoplasia in the majority of animals.

In an investigation of the ovarian changes occurring during treatment of mice with this carcinogen, it was not possible to detect anything other than precocious ageing of the ovaries in the early stages of treatment (Marchant, 1959*b*). There was atrophy of tissue due to rapid loss of follicles, followed by reduction in corpora lutea, with their fusion and disappearance or hyalinisation, and the appearance of pigment. The earliest tumour nodule was detected at the stage in DMBA treatment when follicles and oocytes had almost entirely disappeared and the tumours steadily increased in numbers from then on.

In the experiment reported above, in which normal ovaries were grafted to normal mice whose own ovaries had been removed, histological examination revealed only 2 follicles in 20 ovaries. In most cases the ageing of the ovarian tissue had proceeded to a stage comparable with that reached after several months of DMBA treatment, when a considerable number of tumours or tumour nodules would be found in DMBA-treated mice, but no neoplastic nodules were detected in these grafts of normal ovaries. This suggests that the induction of ovarian tumours in mice treated with DMBA is primarily a result of some phenomonon additional to, or quite apart from, the ageing effects with their consequent changes in hormonal balance which occur after treatment with this carcinogen.

The grafting of normal ovaries did give rise to the development of large fluid-filled cysts in the majority of animals. Further evidence for this was found in another experiment in which normal ovaries were grafted to mice treated with DMBA (Marchant, 1960). Again no trace of solid tumour nodules was found. The appearance of these cysts in a series of ovaries which did not develop any granulosa-celled tumours would appear to exclude them from the same category of growth. They were extremely thin-walled and transparent and, when pierced, they collapsed leaving an amount of tissue no greater than would be found in atrophied non-cystic ovaries. Jones and Krohn (1960) found small fluid-filled cysts in orthotopic ovarian autografts after 25 days or less. They considered them to have arisen from proliferating cells derived from the germinal epithelium.

### SUMMARY

First generation hybrid mice from $C_{57}$Bl mothers and IF fathers were given fortnightly skin paintings of olive oil, each containing 1 mg. of 9 : 10-dimethyl-1 : 2 benzanthracene (DMBA). At intervals of 10, 20, 30, 40 and 60 days from the beginning of treatment, both ovaries were removed from a group of mice and transplanted to the ovarian capsules of untreated hybrids whose own ovaries had been removed. Another group of untreated hybrids received grafts of ovaries from untreated mice.

A high proportion of mice bearing bilateral grafts of ovaries from DMBA-treated mice developed ovarian tumours, most of them being macroscopic. The proportion of mice bearing tumours in each group did not vary greatly with the duration of pretreatment of the ovarian grafts. No ovarian tumours developed in mice grafted with normal ovaries. Large fluid-filled cysts developed in several mice in each group, including those grafted with normal ovaries.

This work was supported by the Birmingham Branch of the British Empire Cancer Campaign.

### REFERENCES

HOWELL, J. S., MARCHANT, J. AND ORR, J. W.—(1954) *Brit. J. Cancer*, **8**, 635.
JONES, E. C. AND KROHN, P. L.—(1960) *J. Endocrin.*, **20**, 135.
MARCHANT, J.—(1959a) *Acta. Un. int. Cancr.*, **15**, 196.
*Idem.*—(1959b) *Brit. J. Cancer*, **13**, 652.
*Idem.*—(1960) *Ibid.*, **14**, 514.